# Hidden prevalence of lower urinary tract symptoms in healthy nulligravid young women

**DOI:** 10.1007/s00192-015-2754-1

**Published:** 2015-06-18

**Authors:** Hendrikje M. K. van Breda, J. L. H. Ruud Bosch, Laetitia M. O. de Kort

**Affiliations:** Department of Urology (610), Radboud University Medical Center, P.O. Box 9101 (610), NL-6500 HB Nijmegen, The Netherlands; Department of Urology (HP C 04.236), University Medical Centre Utrecht, Heidelberglaan 100, 3584 CX Utrecht, The Netherlands

**Keywords:** Lower urinary tract symptoms (LUTS), Nulligravida, Prevalence, Urinary incontinence, Women

## Abstract

**Introduction and hypothesis:**

Lower urinary tract symptoms (LUTS) and urinary incontinence (UI) may have a major impact on quality of life. However, not all individuals with urological complaints seek medical advice. The aim of this study was to investigate the prevalence of LUTS in young otherwise healthy nulligravid women and the accompanying burden.

**Methods:**

A total of 159 young presumably healthy female medical students aged 18–30 years were recruited at their university. All completed the International Consultation on Incontinence Modular Questionnaire for Female Lower Urinary Tract Symptoms. The prevalence of LUTS and the accompanying burden were measured. Correlations between symptoms and bother were analysed using Spearman’s rho.

**Results:**

LUTS was found in 94.3 % of the women, urgency at least sometimes in 14.5 %, and hesitancy in 14.5 %. Nocturia once a night was reported by 18.2 % of the women; none reported nocturia of more than twice a night. Involuntarily loss of urine was reported by 20.1 % of the women but none reported this occurring more than twice a week. The median value of all bother scores was 0; the highest bother score was for urgency. For all questions a positive correlation was found between symptoms and bother; a strong correlation was found for bladder pain, urgency UI, stress UI and overall UI.

**Conclusions:**

In a presumably healthy population of young nulligravid women the prevalence of LUTS and UI was high, but with relatively low bother.

## Introduction

Lower urinary tract symptoms (LUTS) and urinary incontinence (UI) may have a major impact on quality of life. However, not all individuals with urological complaints seek medical advice. The exact prevalence of complaints probably varies with age and sex, and the prevalence of LUTS/UI among young otherwise healthy nulligravid women has not yet been studied extensively. Previously in our centre we have investigated LUTS in young otherwise healthy men, compared with hypospadias patients as a control group, using the International Prostate Symptom Score (IPSS) and found an unexpectedly high prevalence of LUTS: 7 % had moderate to severe LUTS (IPSS >7) [1]. These findings prompted initiation of a comparable study among young nulligravid women.

Knowledge on the prevalence of LUTS in healthy young nulligravid women may be important for establishing what can be considered as ‘normal’ in the general population; this is particularly important in the context of therapeutic studies. Alternatively, there may be a ‘hidden’ health problem requiring attention. Therefore, the present study aimed to establish the prevalence of LUTS in young otherwise healthy nulligravid women and, especially, the accompanying burden.

## Materials and methods

The study was approved by the local ethics committee and written informed consent was obtained from all participants. Between April 2011 and June 2011 young presumably healthy nulligravid female medical students (aged 18–30 years) were recruited at the university by oral announcement of the study. Those interested in participating were invited to our Urology Department. Excluded were females aged <18 or >30 years with a history of urological disease, neurological disease, present symptoms of urinary tract infection (UTI), pregnancy or previous pregnancy.

All subjects were asked to fill in the International Consultation on Incontinence Modular Questionnaire for Female Lower Urinary Tract Symptoms (ICIQ-FLUTS) questionnaire [2]. The questionnaire consists of four questions on bladder filling, three on voiding and five on incontinence. Each question concerning urinary symptoms allows the patient to choose one out of five answers indicating increasing frequency of the particular symptom: ‘never’ (0), ‘occasionally’ (1), ‘sometimes’ (2), ‘most of the time’ (3), and ‘always’ (4). The ICIQ-FLUTS has no scoring system or established cut-off points. We considered a score ≥2 (at least sometimes) as positive for having the symptom. Frequency was defined as more than eight micturitions a day. The total sum score of the ICIQ-FLUTS ranges from 0 to 48 (asymptomatic to very symptomatic). Nocturia of two or more, day time frequency of nine or more, and urgency were considered to be storage symptoms, whereas hesitancy, straining, and intermittency were considered to be voiding symptoms.

Each question on symptoms is linked to a question on bother. There are no established cut-off points for the bother score. All subjects also filled in a questionnaire concerning the exclusion criteria: age, urological history, neurological history, pregnancy or previous pregnancy, present UTI.

Statistical analysis was performed with SPSS version 20. Mean values with standard deviation (SD) and median values with interquartile ranges (IQR) were calculated. Correlations were analysed using Spearman’s rho. A *p* value of <0.05 was considered significant. A correlation coefficient of >0.7 was considered to indicate a strong correlation.

## Results

Initially, 178 students came to the Urology Department to participate. Of these, 19 were excluded: 1 had symptoms of a UTI, 1 had had a pregnancy in the past, 3 were aged ≥30 years, 1 was known to have a neurological disease, and 13 did not fill in the entire questionnaire. The median age of the participants was 22 years (IQR 20–24 years). None of the students had sought medical advice for their urinary symptoms or were planning to do so after filling in the questionnaire.

The results of the ICIQ-FLUTS are presented in Table 1, except those for micturition frequency and nocturia. Nocturia once a night was reported by 29 of the 159 women (18.2 %) and twice a night by 2 (1.3 %); none reported nocturia of more than twice a night. Concerning micturition frequency, 113 women (71.1 %) voided 1 to 6 times a day, 31 (19.5 %) 7 or 8 times a day, 13 (8.2 %) 9 or 10 times a day, and 2 (1.3 %) 11 or 12 times a day. Nine women (5.7 %) had no symptoms at all, i.e. selected ‘never’ as the answer to every question. Urgency and hesitancy were both reported ‘at least sometimes’ (i.e. ‘sometimes’, ‘most of the time’ or ‘always’) by 23 women (14.5 %). Bladder pain was experienced ‘at least sometimes’ by 6 women (3.9 %).

**Table 1:** Results of the ICIQ-FLUTS questionnaire, expressed as frequency of occurrence, in the 159 women included

Score	Bladder pain	Urgency	Hesitancy	Straining	Intermittency	Urinary incontinence			Nocturnal enuresis
						Urgency	Stress	Unexplained	
0 (never)	123 (77.4 %)	51 (32.1 %)	60 (37.7 %)	110 (69.2 %)	124 (78.0 %)	126 (79.2 %)	113 (71.1 %)	156 (98.1 %)	159 (100 %)
1 (occasionally)	30 (18.9 %)	85 53.5 %)	76 (47.8 %)	39 (24.5 %)	33 (20.8 %)	27 (17.0 %)	41 (25.8 %)	3 (1.9 %)	0
2 (sometimes)	6 (3.8 %)	18 (11.3 %)	19 (11.9 %)	8 (5.0 %)	1 (0.6 %)	6 (3.8 %)	5 (3.1 %)	0	0
3 (most of the time)	0	3 (1.9 %)	4 (2.5 %)	1 (0.6 %)	0	0	0	0	0
4 (always)	0	2 (1.3 %)	0	1 (0.6 %)	1 (0.6 %)	0	0	0	0
>0 (any symptom)	36 (22.6 %)	108 (67.9 %)	99 (62.3 %)	49 (30.8 %)	35 (22.0 %)	33 (20.8 %)	46 (28.9 %)	3 (1.9 %)	0
≥2 (at least sometimes)	6 (3.8 %)	23 (14.5 %)	23 (14.5 %)	10 (6.2 %)	2 (1.2 %)	6 (3.8 %)	5 (3.1 %)	0 (0 %)	0 (0 %)
Range	0–2	0–4	0–3	0–4	0–4	0–2	0–2	0–1	
Mean (SD)	0.26 (±0.5)	0.87 (0.8)	0.79 (0.8)	0.39 (0.7)	0.25 (0.5)	0.25 (0.5)	0.32 (0.5)	0.02 (0.1)	0
Median (IQR)	0 (0–0)	1 (0–1)	1 (0–1)	0 (0–1)	0 (0–0)	0 (0–0)	0 (0–1)	0 (0–0)	0

Involuntarily loss of urine was reported by 32 women (20.1 %), by 27 (17.0 %) once a week or less, and by 5 (3.1 %) twice a week. None of the women had involuntary loss of urine more than twice a week. The 32 women with involuntary loss of urine were divided into subgroups according to the type of incontinence. Of these 32 women, 16 (50 %) had both stress and urgency UI, 5 (15.6 %) had urgency UI only, 4 (12.5 %) had stress UI only, and 4 (12.5 %) had urgency UI and unexplained UI. One woman had all three types of UI and 2 (6.2 %) had none. None of the women reported nocturnal enuresis.Urgency, hesitancy, straining and intermittency were reported ‘at least most of the time’ (i.e. ‘most of the time’ or ‘always’) by 5 women (3.1 %), 4 women (2.5 %), 2 women (1.3 %) and 1 woman (0.6 %), respectively. 

The scores for the different categories were summed, i.e. those for storage subscales, and for voiding and incontinence. The median values for the summed scores for storage complaints were 1 (occasionally, IQR 1–2), voiding complaints 1 (occasionally, IQR 1–2), and incontinence 0 (never, IQR 0–1). The median total score was 4/48 (IQR 2–5).

The bother scores are shown in Table 2. For all bother scores the median value was 0. The highest bother score was for urgency. Table 3 shows the bother scores in women with a symptom score of ≥2 (‘at least sometimes’), a symptom score of ≥3 (‘at least most of the time’), nocturia and frequency. Women with incontinence showed the highest bother score.

**Table 2:** Bother scores as measured using the ICIQ-FLUTS questionnaire in the 159 women included

Bother score	Nocturia	Bladder pain	Urgency	Frequency ≥9	Hesitation	Straining	Intermittency	Urinary incontinence				Nocturnal enuresis
								Overall	Urgency	Stress	Unexplained	
0	135 (84.9 %)	121 (76.1 %)	92 (57.9 %)	118 (74.2 %)	119 (74.8 %)	132 (83.0 %)	144 (90.6 %)	135 (84.9 %)	129 (81.1 %)	121 (76.1 %)	156 (98.1 %)	159 (100 %)
1	7 (4.4 %)	13 (8.2 %)	24 (15.1 %)	15 (9.4 %)	22 (13.8 %)	13 (8.2 %)	6 ((3.8 %)	6 (3.8 %)	10 (6.3 %)	13 (8.2 %)	-–	–
2	7 (4.4 %)	13 (8.2 %)	17 (10.7 %)	9 (5.7 %)	9 (5.7 %)	8 (5.0 %)	6 (3.8 %)	3 (1.9 %)	7 (4.4 %)	8 (5.0 %)	2 (1.3 %)	–
3	3 (1.9 %)	5 (3.1 %)	12 (7.5 %)	6 (3.8 %)	3 (1.9 %)	2 (1.3 %)	1 (0.6 %)	3 (1.9 %)	4 (2.5 %)	7 (4.4 %)	–	–
4	2 (1.3 %)	4 (2.5 %)	5 (3.1 %)	2 (1.3 %)	3 (1.9 %)	2 (1.3 %)	1 (0.6 %)	3 (1.9 %)	1 (0.6 %)	4 (2.5 %)	–	–
5	2 (1.3 %)	1 (0.6 %)	2 (1.3 %)	2 (1.3 %)	2 (1.3 %)	1 (0.6 %)	1 (0.6 %)	4 (2.5 %)	1 (0.6 %)	1 (0.6 %)	–	–
6	1 (0.6 %)	1 (0.6 %)	4 (2.5 %)	3 (1.9 %)	1 (0.6 %)	–	–	–	2 (1.3 %)	3 (1.9 %)	–	–
7	2 (1.3 %)	1 (0.6 %)	–	2 (1.3 %)	–	–	–	5 (3.1 %)	3 (1.9 %)	–	–	–
8	–	–	2 (1.3 %)	2 (1.3 %)	–	1 (0.6 %)	–	–	2 (1.3 %)	2 (1.3 %)	1 (0.6 %)	–
9	–	–	–	–	–	–	–	–	–	–	–	–
10	–	–	1 (0.6 %)	–	–	–	–	–	–	–	–	–
>0	24 (15.1 %)	38 (23.9 %)	67 (42.1 %)	41 (25.8 %)	40 (25.2 %)	27 (17.0 %)	15 (9.4 %)	24 (15.1 %)	30 (18.9 %)	38 (23.9 %)	3 (1.9 %)	0
Range	0–7	0–7	0–10	0–8	0–6	0–8	0–5	0–7	0–8	0–8	0–8	
Mean (SD)	0.43 (1.3)	0.55 (1.2)	1.09 (1.8)	0.74 (1.7)	0.48 (1.1)	0.35 (1.0)	0.19 (0.7)	0.55 (1.6)	0.59 (1.6)	0.66 (1.5)	0.08 (0.7)	0
Median (IQR)	0 (0–0)	0 (0–0)	0 (0–0)	0 (0–1)	0 (0–1)	0 (0–0)	0 (0–0)	0 (0–0)	0 (0–0)	0 (0–0)	0 (0–0)	0

**Table 3:** Bother scores in women with a symptom score of ≥2 (i.e. ‘sometimes’ or more frequently), a symptom score of ≥3 (i.e. ‘most of the time’ or ‘always’), nocturia once or more a night, nocturia twice or more a night, frequency nine times or more a day, and frequency 11 times or more a day, as measured by the ICIQ-FLUTS questionnaire

	Nocturia (times a night)		Bladder pain		Urgency		Frequency (times a day)		Hesitation		Straining		Intermittency		Urinary incontinence								Nocturnal enuresis	
															Overall		Urgency		Stress		Unexplained			
	≥1	≥2	≥2	≥3	≥2	≥3	≥9	≥11	≥2	≥3	≥2	≥3	≥2	≥3	≥2	≥3	≥2	≥3	≥2	≥3	≥2	≥3	≥2	≥3
No. of women	31	2	6	0	23	5	15	2	23	4	10	2	2	1	5	0	6	0	5	0	0	0	0	0
Bother score																								
Range	0–7	0–7	1–7	–	0–10	3–10	0–8	2–8	0–6	0–6	0–8	0–8	0–4	–	5–7	–	3–8	–	1–8	–	–	–	–	–
Mean (SD)	1.9 (2.2)	3.5 (5.0)	3.2 (2.6)	–	3.6 (2.9)	6.6 (2.6)	3.5 (2.8)	5 (4.2)	1.5 (1.9)	3 (2.9)	2.0 (2.6)	4 (5.7)	2.0 (2.8)	0	6.6 (0.9)	–	6.5 (1.9)	–	5.2 (3.1)	–	–	–	–	–
Median (IQR)	1 (0–3)	–	2 (1–6.3)	–	3 (1–6)	6 (4.5–9)	3 (0–6)	–	1 (0–3)	3 (0.3–5.8	1 (0–4)	4		–	7 (6–7)	–	7 (5.3–8)	–	6 (2–8)	–	–	–	–	–

For all questions a positive correlation was found between symptoms and bother. A strong correlation (>0.7) was found for bladder pain, urgency UI, stress UI and overall UI (Table 4).

**Table 4:** Correlation between symptoms and bother measured with Spearman’s rho

Nocturia ≥2 times a night		Bladder pain		Urgency		Frequency ≥9		Hesitation		Straining		Intermittency		Urinary incontinence								Nocturnal enuresis
														Overall		Urgency		Stress		Unexplained		
Spearman’s rho	*p* value	Spearman’s rho	*p* value	Spearman’s rho	*p* value	Spearman’s rho	*p* value	Spearman’s rho	*p* value	Spearman’s rho	*p* value	Spearman’s rho	*p* value	Spearman’s rho	*p* value	Spearman’s rho	*p* value	Spearman’s rho	*p* value	Spearman’s rho	*p* value	
0.68	<0.01	0.93	<0.01	0.63	<0.01	0.40	<0.01	0.41	<0.01	0.61	<0.01	0.61	<0.01	0.85	<0.01	0.91	<0.01	0.88	<0.01	0.66	<0.01	–

## Discussion

In this group of young healthy nulligravid women the vast majority (94.3 %) reported some kind of LUTS or UI; only 5.7 % of women had no symptoms at all. Furthermore, 40.9 % of the group had one or more complaints at least sometimes. Comparing these results with those of similar studies among young women, more women had incontinence, urgency, hesitancy and straining, but fewer had nocturia, frequency and intermittency [3–9]. However, the results of previous studies are not consistent. For example, the reported prevalence of UI ranges from 3.4 % to 20.1 %, urgency from 3 % to 19.5 %, hesitancy from 9.3 % to 14.1 %, straining from 1.5 % to 14.1 %, nocturia from 9.5 % to 9 %, frequency from 7 % to 46.1 % and intermittency from 1.6 % to 31.5 % [3–9]. In the present study, LUTS was defined as being present when it was reported to occur ‘at least sometimes’. In other studies different definitions/thresholds, different surveys and different administration techniques have been used. For example, Liao et al. defined the presence of LUTS as the self-reported occurrence during the past 12 months, using a self-developed (paper) questionnaire [6]. Irwin et al. asked whether or not symptoms were experienced using a self-developed questionnaire; their subjects were interviewed by telephone [5]. Chuang and Kuo used the IPSS questionnaire and face-to-face interviews with their participants [3]. However, the grade of symptom severity was often not adequately described or differed between these studies. Moreover, the use of different questionnaires, definitions and methodologies make comparison with other studies and interpretation of results difficult. Furthermore, the populations differed between studies regarding age, ethnicity and social background.

In the present study 9.5 % of the women had a daytime frequency of ≥9, whereas others have reported subject experiencing frequency ranging from 7–57.1 % [3, 5–7, 9]. However, these results should be interpreted with caution because no information on daily fluid intake was available in the present nor in the other studies. In this study, UI of any type and frequency were reported by 20.1 % of women (with 3.1 % experiencing urinary loss at least twice a week), whereas in other studies UI of any type and frequency have been reported by 3.4–20.1 % of women [4–9]. However, definitions may have been different between the studies. In the present study, slightly more women reported urgency UI than stress UI; this might be because the women were aged ≤30 years and nulligravid. A study among young nulligravid women conducted by O’Halloran et al. revealed the opposite: slightly more women reported stress UI than urgency UI [8]. A remarkable finding is that two women who reported UI at least sometimes (more often than never), reported that they never experienced the different types of UI when differentiated into subgroups; we have no clear explanation for this finding.

In this study, 18.2 % of the women reported nocturia once a night and 1.3 % twice a night; none reported nocturia more than twice a night. The clinical relevance of only one episode of nocturia per night is yet to be determined. However, given the large decrease in prevalence of nocturia when the definition was changed from at least one micturition to two or more micturitions per night, suggests that one micturition per night is part of the normal spectrum. Other studies comparing nocturia with bother have shown that one micturition per night does not identify persons with bothersome nocturia and thus is not a suitable criterion for clinically relevant nocturia [10, 11].

In the present study, for all questions a positive correlation was found between symptoms and bother, most strongly between bladder pain and urgency UI. Bother scores were highest in women experiencing incontinence and storage symptoms at least sometimes, whereas those with voiding symptoms had relatively low bother scores. The presence of urgency UI had slightly more influence on bother scores than stress UI. It has been reported that women with UI and overactive bladder symptoms show lower physical and mental health scores than women without these symptoms [8, 12]. Studies of the effects of UI on the quality of life have shown that mixed UI is more bothersome than urgency UI or stress UI alone, and that urgency UI alone is more bothersome than stress UI alone [12–15].

In general, the women in this study had higher symptom scores than bother scores; this might imply that their perceived symptoms were not always annoying but may have been interpreted as physiological. In the present study, if a symptom was scored as ‘sometimes’ or more frequently, that symptom was defined as (relevantly) present. Choosing another threshold, for example ‘most of the time’, lowers the prevalence. In our opinion, a symptom occurring ‘sometimes’ is relevant. And as expected, if a symptom occurred more often, the bother score was higher. However, as there is no established cut-off point for the ICIQ questionnaires, this issue remains debatable.

The present study population consisted of females not seeking medical advice for LUTS, so the question arises as to whether a symptom should be called a complaint or whether it should be considered as ‘normal’. The fact that the bother scores were relatively low indicates that not all symptoms were complaints. On the other hand, reluctance and shame may play a role in women not seeking medical advice.

The results of the present study should be viewed within the context of its limitations. First, only medical students were invited to participate, mainly for logistical reasons. This group may not be representative of the general population, mainly because of a higher educational level and (perhaps) social values that might differ from those of the general population. O’Halloran et al. [8] found that in a population of young nulligravid women, incontinence was slightly more common in students than in nonstudents. In our group of medical students, some additional knowledge/training in urology and awareness of urological signs and symptoms can be assumed. These factors may have influenced the outcomes. A limitation concerning other factors that could have influenced LUTS is that we had no information about sexual activity, BMI and smoking or drug use. Another limitation is the use of self-reporting to measure LUTS. There is evidence that self-reports are vulnerable to inaccuracy relative to the criterion standard of a physician diagnosis based on assessment of patient history and urodynamic evaluation [16, 17]. However, the use of physician diagnosis would have introduced a degree of subjectivity and changes on the bother scale. Also, our results might have differed slightly had information been collected by telephone, mail, or via face-to-face interviews [18, 19]. In addition, because we recruited participants via an announcement in the medical school, no information was available on the response rate. Some individuals who were invited declined to participate, possibly creating some selection bias. We have no further information on the candidates who did not participate.

In conclusion, in this presumably healthy group of young nulligravid women the prevalence of LUTS and UI was high, although with relatively low bother. These findings should be taken into account in therapeutic studies in a comparable age group. Furthermore, the presence of LUTS or UI in this study population appears to be no reason to seek medical advice.

## References

[1] Rynja SP, Wouters GA, Van Schaijk M, Kok ET, De Jong TP, De Kort LM (2009). Long-term followup of hypospadias: functional and cosmetic results. J Urol.

[2] Jackson S, Donovan J, Brookes S, Eckford S, Swithinbank L, Abrams P (1996). The Bristol female lower urinary tract symptoms questionnaire: development and psychometric testing. Br J Urol.

[3] Chuang FC, Kuo HC (2010). Prevalence of lower urinary tract symptoms in indigenous and non-indigenous women in Eastern Taiwan. J Formos Med Assoc.

[4] Hansen BB, Svare J, Viktrup L, Jorgensen T, Lose G (2012). Urinary incontinence during pregnancy and 1 year after delivery in primiparous women compared with a control group of nulliparous women. Neurourol Urodyn.

[5] Irwin DE, Milsom I, Hunskaar S, Reilly K, Kopp Z, Herschorn S, Coyne K, Kelleher C, Hampel C, Artibani W, Abrams P (2006). Population-based survey of urinary incontinence, overactive bladder, and other lower urinary tract symptoms in five countries: results of the EPIC study. Eur Urol.

[6] Liao YM, Dougherty MC, Biemer PP, Boyington AR, Liao CT, Palmer MH, Lynn MR (2007). Prevalence of lower urinary tract symptoms among female elementary school teachers in Taipei. Int Urogynecol J.

[7] Liao YM, Yang CY, Kao CC, Dougherty MC, Lai YH, Chang YM, Chen HL, Chang LI (2009). Prevalence and impact on quality of life of lower urinary tract symptoms among a sample of employed women in Taipei: a questionnaire survey. Int J Nurs Stud.

[8] O'Halloran T, Bell RJ, Robinson PJ, Davis SR (2012). Urinary incontinence in young nulligravid women: a cross-sectional analysis. Ann Intern Med.

[9] Pinnock CB, Marshall VR (1997). Troublesome lower urinary tract symptoms in the community: a prevalence study. Med J Aust.

[10] Tikkinen KAO, Johnson TM, Tammela TLJ, Sintonen H, Haukka J, Huhtala H, Auvinen A (2010). Nocturia frequency, bother, and quality of life: how often is too often? A population-based study in Finland. Eur Urol.

[11] Weiss JP, Wein AJ, van Kerrebroeck P, Dmochowski R, Fitzgerald M, Tikkinen KAO, Abrams P (2011). Nocturia: new directions. Neurourol Urodyn.

[12] Chiaffarino F, Parazzini F, Lavezzari M, Giambanco V, Grp Interdisciplinare Studio I (2003). Impact of urinary incontinence and overactive bladder on quality of life. Eur Urol.

[13] Coyne KS, Zhou Z, Thompson C, Versi E (2003). The impact on health-related quality of life of stress, urge and mixed urinary incontinence. BJU Int.

[14] Milsom I, Kaplan SA, Coyne KS, Sexton CC, Kopp ZS (2012). Effect of bothersome overactive bladder symptoms on health-related quality of life, anxiety, depression, and treatment seeking in the United States: results from EpiLUTS. Urology.

[15] Minassian VA, Devore E, Hagan K, Grodstein F (2013). Severity of urinary incontinence and effect on quality of life in women by incontinence type. Obstet Gynecol.

[16] Kirschner-Hermanns R, Scherr PA, Branch LG, Wetle T, Resnick NM (1998). Accuracy of survey questions for geriatric urinary incontinence. J Urol.

[17] Sandvik H, Hunskaar S, Vanvik A, Bratt H, Seim A, Hermstad R (1995). Diagnostic classification of female urinary-incontinence – an epidemiologic survey corrected for validity. J Clin Epidemiol.

[18] Bowling A (2005). Mode of questionnaire administration can have serious effects on data quality. J Publ Health.

[19] Rhodes T, Girman CJ, Jacobsen SJ, Guess HA, Hanson KA, Oesterling JE, Lieber MM (1995). Does the mode of questionnaire administration affect the reporting of urinary symptoms. Urology.

